# Direct identification of antibiotic resistance genes on single plasmid molecules using CRISPR/Cas9 in combination with optical DNA mapping

**DOI:** 10.1038/srep37938

**Published:** 2016-12-01

**Authors:** Vilhelm Müller, Fredrika Rajer, Karolin Frykholm, Lena K. Nyberg, Saair Quaderi, Joachim Fritzsche, Erik Kristiansson, Tobias Ambjörnsson, Linus Sandegren, Fredrik Westerlund

**Affiliations:** 1 Department of Biology and Biological Engineering, Chalmers University of Technology, Gothenburg, Sweden; 2 Department of Medical Biochemistry and Microbiology, Uppsala University, Uppsala, Sweden; 3 Department of Astronomy and Theoretical Physics, Lund University, Lund, Sweden; 4 Department of Applied Physics, Chalmers University of Technology, Gothenburg, Sweden; 5 Department of Mathematical Sciences, Chalmers University of Technology/University of Gothenburg, Gothenburg, Sweden; 6 Centre for Antibiotic Resistance Research (CARe), University of Gothenburg, Gothenburg, Sweden

## Abstract

Bacterial plasmids are extensively involved in the rapid global spread of antibiotic resistance. We here present an assay, based on optical DNA mapping of single plasmids in nanofluidic channels, which provides detailed information about the plasmids present in a bacterial isolate. In a single experiment, we obtain the number of different plasmids in the sample, the size of each plasmid, an optical barcode that can be used to identify and trace the plasmid of interest and information about which plasmid that carries a specific resistance gene. Gene identification is done using CRISPR/Cas9 loaded with a guide-RNA (gRNA) complementary to the gene of interest that linearizes the circular plasmids at a specific location that is identified using the optical DNA maps. We demonstrate the principle on clinically relevant extended spectrum beta-lactamase (ESBL) producing isolates. We discuss how the gRNA sequence can be varied to obtain the desired information. The gRNA can either be very specific to identify a homogeneous group of genes or general to detect several groups of genes at the same time. Finally, we demonstrate an example where we use a combination of two gRNA sequences to identify carbapenemase-encoding genes in two previously not characterized clinical bacterial samples.

The rapid increase of bacteria resistant to antibiotics imposes a major threat to human health and threatens to negate much of modern medicine in a near future^1^. In combination with the absence of new antibiotic treatment alternatives, one of the main problems is a lack of tools for rapid point-of-care diagnostics. With fast and robust diagnostics of resistance, adequate treatment could be administered directly, existing antibiotics could be used more efficiently and “last resort” antibiotics could be spared^2^. A key reason for the rapid spread of antibiotic resistance is horizontal transfer of resistance genes located on mobile genetic elements, such as plasmids^3^. Recently, plasmid-mediated resistance to last resort antibiotics, such as carbapenems^4^ and polymyxins^5^, have been reported. A method allowing for fast characterization of plasmids and their resistance genes is therefore highly desirable in order to enable more efficient antibiotic treatment, minimize morbidity and prevent the rapid spread of resistant bacteria. Such a method could furthermore be a useful research tool in plasmid biology to understand the fundamental principles of plasmid epidemiology and evolution.

Current methods to characterize plasmids include S1-coupled pulsed field gel electrophoresis (PFGE) for plasmid number and sizing^6^, conjugation-based methods for single plasmid studies and PCR-based or hybridization-based methods for detection of genes and plasmid types^7^. All these methods suffer from being slow (requiring days or weeks for completion for S1/PFGE and conjugational approaches) and/or require existing knowledge of the targeted sequence (as for PCR-based or hybridization-based methods). These methods are now increasingly being replaced by next generation DNA sequencing approaches^8^, providing basepair resolution. Despite the improvement of sequencing techniques during the last decades, assembling plasmid sequences is not trivial due to their dynamic and repetitive nature which often requires time-consuming downstream bioinformatics analysis to obtain the complete picture^3^. Furthermore, whole genome sequencing is so far dependent on bacterial cultivation, DNA preparation, sequencing library preparation and long data collection procedures and is therefore slow, hampering its use in rapid diagnostics. A comparison of different plasmid analysis methods can be found in a recent review^3^.

Optical DNA mapping is based on visualizing large size DNA molecules at the single DNA molecule level using fluorescence microscopy, providing coarse-grained sequence information when stretched either on surfaces^9^, or in nanofluidic devices^10^,^11^. We have previously developed a single-step optical DNA mapping assay, that is based on competitive binding between the fluorescent dye YOYO-1 (YOYO) and the AT-selective molecule netropsin, both commercially available at low cost^12^,^13^. Netropsin competes with YOYO for AT-rich regions, meaning that the DNA molecule will have an overall lower emission from AT-rich regions than GC-rich regions. The variation in emission intensity can be visualized on DNA stretched in nanochannels and thereby a barcode reflecting the underlying sequence is formed along the DNA. This assay has allowed us to trace how previously uncharacterized plasmids spread during a nosocomial outbreak^14^ as well as identify plasmids from a database containing all known plasmid DNA sequences^15^. One main advantage with the technique for plasmid analysis is that intact plasmids can be directly visualized in the channels and hence we can easily discard any remaining chromosomal DNA or fragmented plasmids^16^.

The main limitation with the assay in its current format is that there is no direct indication that a certain resistance gene is present on a specific plasmid. McCaffrey *et al*. recently demonstrated that specific genes can be visualized in optical DNA maps using CRISPR/Cas9^17^. The use of Cas9 for gene editing has exploded in the last years due to its versatility and specificity^18^. In short, the enzyme uses a 20 basepair (bp) RNA-sequence to direct the Cas9 enzyme to a specific sequence and cut the DNA backbone on both strands at this location. This 20 bp sequence is part of the guide RNA (gRNA) that binds to Cas9 to guide it to the correct sequence. Synthetically, the gRNA can be divided into two fragments, tracrRNA and crRNA, where tracrRNA always has the same sequence and binds the gRNA to Cas9, while the crRNA guides the Cas9 to the sequence of interest. The sequence of the crRNA can be chosen almost freely; the only requirement is that it is followed by a three base sequence (NGG) called the PAM-sequence, making the full recognition sequence 23 bases long. Since the PAM-sequence occurs frequently in most DNA sequences, the Cas9 can be directed to almost any gene with very high specificity. McCaffrey *et al*. used a mutated version of Cas9 (D10A) that only cuts one of the strands and subsequently repaired the nick(s) with a fluorescent nucleobase to visualize specific genes on DNA stretched in nanochannels^17^.

We here use wild-type Cas9 to detect resistance genes in bacterial plasmids. Cas9 with a crRNA targeting a particular resistance gene is used to cut plasmids into their linear configuration. We then stain the DNA with YOYO and netropsin and visualize where the double-strand breaks (dsbreaks) occurs along the barcodes by stretching the linearized plasmids in nanochannels. If a majority of the dsbreaks occur at the same position along the barcode, the targeted sequence is present on the plasmid (Fig. 1a). We demonstrate the assay on several important resistance genes including the extended spectrum beta-lactamase (ESBL) gene family *bla*_CTX-M_ (group 1 and group 9), and the carbapenemase gene families *bla*_NDM_ and *bla*_KPC_. These groups of beta-lactamases represent the currently largest clinical problem among enteric bacteria and are therefore essential resistance genes to screen for in a clinical situation. We present studies on the clinically relevant case where there is more than one plasmid present in each isolate and also discuss how to confirm that the results are statistically valid. The assay has potential applications ranging all the way from fundamental plasmid biology to epidemiological tracing of plasmids and clinical diagnostics. We discuss how the assay can be optimized for clinical use by combining several gRNAs that identify different genes that give resistance to the same group of antibiotics. Importantly, the assay does not require any additional labeling apart from YOYO and netropsin, and the complexity of the sample preparation, data collection and data analysis compared to the optical mapping assay in its original format is kept at a minimum. We have thus integrated several important parameters that are traditionally used for plasmid identification, including size determination and gene identification, in one single assay that furthermore gives a fingerprint of the plasmid that can be used for further plasmid characterization and tracing (Fig. 1b).

## Results

**Figure 1:**
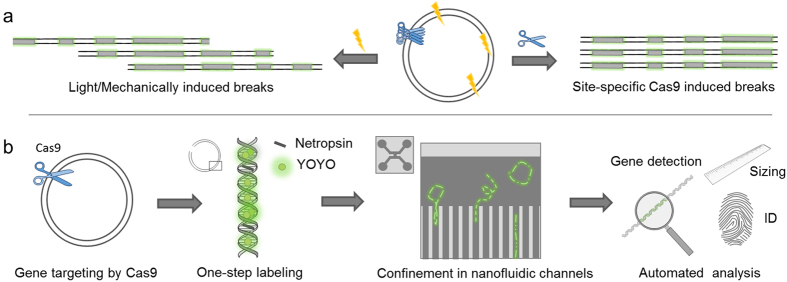
Schematic overview of the Cas9 assay. (**a**) Schematic illustration of the principle of the Cas9 assay. While the Cas9 enzyme (scissors), loaded with a crRNA targeting the gene of interest, will break the circular plasmid at a specific location, a break caused by light or mechanical stress (lightning) will occur anywhere along the contour and the linear barcodes will be circularly permutated. This leads to that for Cas9 (right) all linear fragments will have, within the experimental noise, identical barcodes, while for plasmids broken by light or mechanically, the barcodes will be circularly permutated (left). (**b**) Schematic illustration of the process of the assay. In the first step the DNA is cut by the Cas9 enzyme loaded with crRNA targeting the gene of interest. In the second step, netropsin and YOYO are added to the plasmid sample. In the third step, the plasmids are stretched in nanochannels and the barcode is visualized using fluorescence microscopy. The size of all plasmids, a corresponding barcode ID, a “fingerprint”, for identification and tracing, as well as the presence or absence of a specific gene is obtained in a single experiment.

In this study we use Cas9 to identify resistance genes by cutting circular plasmids into their linear configuration. In our previous studies we cut the plasmids with light to form a linear configuration and visualize the barcode; these dsbreaks appear randomly along the barcode and hence the barcodes obtained are circularly permutated with respect to each other^15^. When Cas9 cuts the plasmid at a specific location, corresponding to a specific gene, a vast majority of the plasmids will be linearized at the *same* location along the barcode and this is directly visible in the barcodes (Fig. 1a and Methods). To confirm the presence of a plasmid of a certain size, we intentionally did not run the reaction to completion, so that some plasmids remained circular. This is important because we can undisputedly confirm that plasmids of a certain size are present in the sample if we detect intact circular plasmids of the corresponding size^16^,^19^. Only linear fragments of a size corresponding to that of a circular plasmid detected are used in the analysis below.

### Detection of resistance genes using CRISPR-Cas9

**Figure 2:**
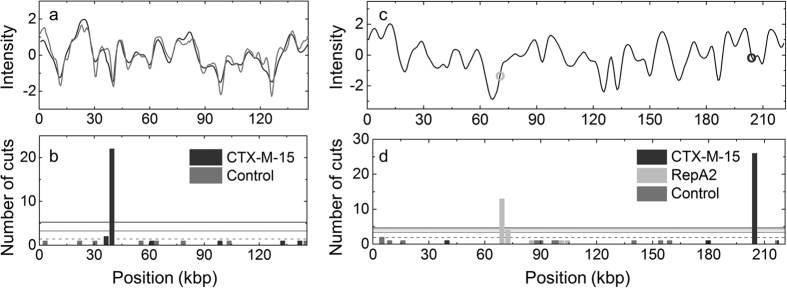
Detection of the *bla*_CTX-M-15_ gene in two samples. (**a**) Consensus barcode for plasmid pEC019 cut either with light (control, gray) or with Cas9 targeting the *bla*_CTX-M-15_ gene (dark gray). (**b**) Histogram showing the location of the dsbreaks in the control sample (gray) and the sample treated with Cas9 targeting the *bla*_CTX-M-15_ gene (dark gray). Using the Cas9, a vast majority of the dsbreaks appear at the same location, showing that the *bla*_CTX-M-15_ gene is present. (**c**) Theoretical barcode for plasmid pUUH239.2 where the circles indicate the presence of the *bla*_CTX-M-15_ gene (dark gray) and the *repA2* gene (light gray), respectively. (**d**) Histogram showing the location of the dsbreaks in the control sample (gray) and the sample treated with Cas9 targeting the *bla*_CTX-M-15_ gene (dark gray) or the *repA2* gene (light gray). Using the Cas9, a vast majority of the cuts appear at the predicted locations, showing that the genes are present. The horizontal lines in the histograms correspond to the mean value for the control experiments (dashed line) and three standard deviations above the mean for all experiments (solid lines) from the balls-in-boxes statistics.

In order to test the applicability of the Cas9 assay we demonstrate that we can identify the clinically most prevalent ESBL gene in Europe, *bla*_CTX-M-15,_ in two previously well-characterized clinical plasmids, pEC019 and pUUH239.2. Plasmid pEC019 has previously been characterized by traditional methods such as S1/PFGE and whole genome sequencing^20^. By S1/PFGE the isolate was shown to contain one plasmid with an estimated size of 150 kbp (+/−5 kbp) and the technique required four full working days for completion. Figure 2a shows consensus barcodes (see Methods) for plasmid pEC019 cut with Cas9 carrying a crRNA designed to recognize the *bla*_CTX-M-15_ gene and the corresponding control experiment where the plasmids are linearized with light. The assay yields, within a couple of hours (from sample loading to results), the size of the plasmid, 146 kbp (+/−4 kbp), in excellent agreement with the previously published estimated size (Supplementary Information, Figure S2)^20^. Figure 2b shows the location of each dsbreak along the barcodes when Cas9 is used (28 plasmid molecules) and when the plasmids are linearized with light (8 plasmid molecules), respectively. While the control experiment has dsbreaks spread along the contour, the Cas9 treated sample has a vast majority of dsbreaks (22 of 28, 79%) at the same location along the barcode, suggesting that they have been cut by the Cas9 enzyme. That 100% of the dsbreaks do not occur at the same location when Cas9 is used can be attributed to plasmids that are either linearized during sample preparation or by light before entering the nanochannels.

To judge whether the fraction of plasmids cut at a specific position is statistically significant we use a simple “balls-in-boxes” approach (details in Methods and Supplementary Methods) to determine how many dsbreaks that can be expected by chance at a certain location. We then set a threshold at this mean value plus three standard deviations as a limit for positive detection. For the pEC019 plasmid in Fig. 2b this threshold ends up at 5.2 (3.22 + 3 × 0.66) dsbreaks when Cas9 is used, far below the detected 22 found in the bin with the most dsbreaks. For the control, only one dsbreak is observed for each position, compared to the threshold of 3.2 (1.55 + 3 × 0.55), confirming that the linearization is random when induced by light.

For the second plasmid, pUUH239.2 (pUUH), the complete sequence of the plasmid has been determined^21^. Therefore, the theoretical barcode can be predicted from the sequence^13^ and we can predict exactly where along the barcode specific genes are located (Fig. 2c). We used the same crRNA, targeting the *bla*_CTX-M-15_ gene, to cut the DNA and 90% of the dsbreaks (26 of 29) appear at the predicted location (Fig. 2d). The assay is general and thus not limited to resistance genes. In Fig. 2c,d we performed an experiment where we cut the pUUH plasmid with a Cas9 targeting *repA2*, a gene that belongs to the replication machinery of the plasmid and is generally assessed in classical replicon typing of plasmids^22^. The cut by Cas9 again occurs at the predicted location along the sequence of the plasmid, indicating that the gene is present and that the plasmid indeed belongs to the IncFII group as known from the earlier study^21^.

We can thus in two consecutive experiments both determine the presence of a specific resistance gene and determine the incompatibility group of the plasmid. In addition, we directly obtain the size of the plasmid, also in this case in perfect agreement with reported values (Supplementary Information, Figure S2), and a fingerprint that can be used for plasmid identification. All experimental consensus barcodes can be found in the Supplementary Information, Figure S3.

### Design of crRNA

**Figure 3:**
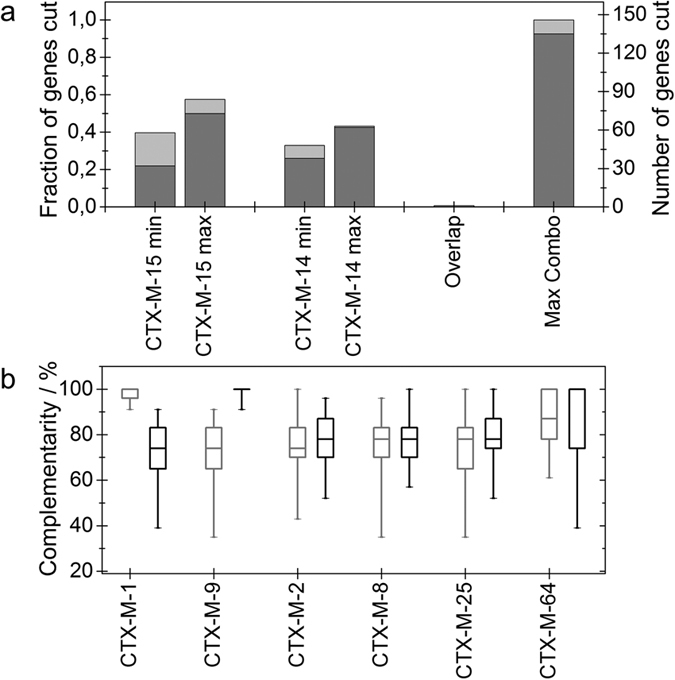
In silico analysis of all resistance genes in the *bla*_CTX-M_ gene family. (**a**) The minimum and maximum number of genes that have a perfect match (dark gray) with crRNAs designed for *bla*_CTX-M-14_ (group 9) and *bla*_CTX-M-15_ (group 1), respectively. Shown is also the overlap between group 1 (*bla*_CTX-M-15_) and group 9 (*bla*_CTX-M-14_) and the number of *bla*_CTX-M_ genes that are possible to target using a combination (combo) of one crRNA designed for group 1 and one designed for group 9. In light gray the fraction of genes with a single mismatch are shown. (**b**) Box diagram showing how well crRNAs, designed to be fully complementary to *bla*_CTX-M-15_ (group 1, gray) and *bla*_CTX-M-14_ (group 9, black), overlap with other *bla*_CTX-M_ genes. The first five genes selected belong to the five major *bla*_CTX-M_ groups. The sixth gene, *bla*_CTX-M-64_ was selected because it is a hybrid of group 1 and group 9^25^. The box shows the median, the upper and lower quartiles and the whiskers ranging from the minimum to the maximum value.

The design of the crRNA is crucial for the assay and different information about the gene content in a specific sample can be obtained by varying the crRNA. We demonstrate this via an *in silico* analysis of the *bla*_CTX-M_ gene family (Fig. 3, Supplementary Tables ST1and ST2). Above we selected specific crRNAs to target the *bla*_CTX-M-15_ gene, however, since the only requirement for the sequence of the crRNA is that it ends with the PAM-motif (NGG), each gene can be targeted at multiple locations. Since many resistance genes occur in groups that often have very similar sequences, the information that can be obtained is different depending on the crRNA selected, which is a useful property of the assay.

In Fig. 3 we use the *bla*_CTX-M_ gene family to demonstrate how the crRNA sequence can be selected to maximize the information obtained from each experiment. The *bla*_CTX-M_ gene family currently contains 146 different reported gene variants that can be phylogenetically divided into five main groups. The divergence within each group can be attributed to antibiotic selection pressure and different individual ESBLs can therefore have different catalytic spectra against beta-lactam antibiotics^23^. The two clinically most widespread groups are group 1 and group 9, each containing approximately 50 genes. Since the sequences of all 146 *bla*_CTX-M_ genes are known, it is straightforward to predict which genes that will be targeted by a specific crRNA (see Methods). For example, based on the sequence of the common *bla*_CTX-M-15_ gene from group 1, we can design 102 possible crRNA sequences that will target this particular gene. Our *in silico* analysis shows that when comparing these 102 crRNA sequences to all available *bla*_CTX-M_ gene sequences (see Methods), each possible crRNA had a perfect match to 32 to 73 (average 52.5) of the *bla*_CTX-M_ genes (Fig. 3a). When performing this analysis, it is important to remember that Cas9 can potentially also cut genes with a one base mismatch in the sequence, in particular if the mismatch is far from the PAM sequence, while for two mismatches the enzyme does not cut in approximately 96% of the cases^24^.

Analogously, for group 9, the other large *bla*_CTX-M_ group, it is possible to, using *bla*_CTX-M-14_ as a starting point, design crRNAs that target 38 to 62 *bla*_CTX-M_ genes (Fig. 3a). Interestingly, the assay shows high specificity, and there is no overlap between group 1 and group 9, *i.e.* no crRNA designed for group 1 targets genes from group 9 and vice versa. From a diagnostics perspective the most important question to answer will be if the isolate collected carries a *bla*_CTX-M_ gene, not primarily which group that the gene belongs to. Our analysis reveals that the vast majority, 135 of the 146 *bla*_CTX-M_ genes, can be targeted by combining one crRNA from group 1 and one from group 9 (Fig. 3a), while the remaining 11 have a single mismatch, which means that they are also potentially targeted by the crRNA.

Figure 3b shows the sequence overlap between crRNAs designed for *bla*_CTX-M-15_ and *bla*_CTX-M-14_ compared to one gene belonging to each of the five main groups, as well as one minor group, and the same information is easily accessible for any known gene variant. A perfect match means that the crRNA will identify the two genes but not separate them, while a match with lower sequence similarity means that the crRNA will discriminate the two genes. Exemplifying the kind of information that can be obtained, we see that almost all crRNAs designed for *bla*_CTX-M-15_ will also target *bla*_CTX-M-1,_ but it is possible to design one specific crRNA that has two mismatches for *bla*_CTX-M-1_ and hence can be used to discriminate the two. For all other gene families, it is straight forward to design an RNA that targets *bla*_CTX-M-15_ but not the other group and the same is true for *bla*_CTX-M-14_. *bla*_CTX-M-64_ is interesting since it is a hybrid of group 1 and group 9^25^. This means that several crRNAs that are designed for either *bla*_CTX-M-14_ or *bla*_CTX-M-15_ will target also *bla*_CTX-M-64_. In this case it will be possible to distinguish *bla*_CTX-M-14,_ *bla*_CTX-M-15_ and *bla*_CTX-M-64_ by running two parallel reactions, one with *bla*_CTX-M-14_ crRNA and one with *bla*_CTX-M-15_ crRNA, both designed to detect *bla*_CTX-M-64_.

### Isolate with more than one plasmid

**Figure 4:**
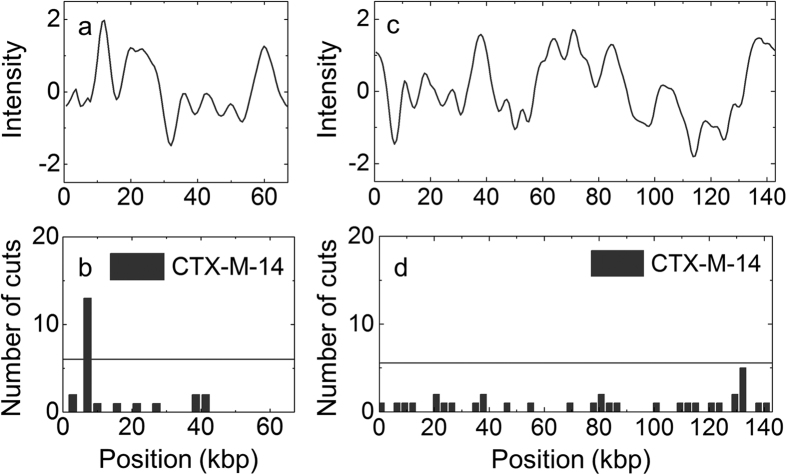
Identification of the *bla*_CTX-M-14_ gene in a sample with multiple plasmids. (**a**) and (**c**) Consensus barcodes for plasmid pEC005A (**a**) and pEC005B (**c**) in presence of Cas9 targeting the *bla*_CTX-M-14_ gene. (**b**) and (**d**) Histograms showing the location of dsbreaks in presence of Cas9 targeting the *bla*_CTX-M-14_ gene. The horizontal lines in the histograms correspond to three standard deviations above the mean from the balls-in-boxes statistics. The results show that the Cas9 has caused a specific dsbreak on plasmid pEC005A and not on pEC005B, showing that the gene is located on the pEC005A plasmid.

In Fig. 4 we show that the assay presented is suitable to identify which plasmid that carries a specific gene in a bacterial isolate containing more than one plasmid. Bacterial isolates of clinical origin often carry more than one plasmid^26^ and it is not straightforward to determine, for example with PCR, which resistance genes that are encoded by which plasmid. We applied the Cas9-assay to isolate ECO-005 that has previously been characterized using traditional techniques^20^, and optical mapping^15^. This isolate has been shown to carry a *bla*_CTX-M-14_ gene and contains two different plasmids, one 67 kbp (pEC005A) and one 139 kbp (pEC005B)^20^. Using the analysis above we designed a crRNA that targets all members of group 9 (to which *bla*_CTX-M-14_ belongs) and no other *bla*_CTX-M_ genes. This crRNA was then used with Cas9 to cut the plasmids in isolate ECO-005, yielding the histograms shown in Fig. 4b and d. Our assay directly demonstrated that the *bla*_CTX-M-14_ gene is present on the small plasmid, and not the large plasmid, in agreement with the previous findings^20^. Simultaneously, we determined the size of both plasmids, in good agreement with the published sizes (Supplementary Information, Figure S2) and consensus barcodes of each plasmid. Importantly, it is also possible to obtain the consensus barcode for the plasmid that does not carry the targeted gene, even though Cas9 did not cut that plasmid. For traditional characterization methods to yield the same results, the coupled S1/PFGE was followed by Southern blotting to reveal which plasmid encoded the gene, adding a further day of work to the previous four, making this kind of analysis take a full working week^20^.

### Detecting carbapenem resistance

Carbapenems are broad-spectrum beta-lactam antibiotics commonly used as a last resort antibiotic treatment option for many forms of multidrug resistant bacteria. The prevalence of resistance towards carbapenems is however rapidly increasing throughout the world^4^. It is therefore necessary to rapidly identify the presence of carbapenemases, beta-lactamases with catalytic activity against carbapenems, to avoid treating patients with ineffective antibiotics. There are several different groups of carbapenemases, two of the clinically most important ones are the *bla*_KPC_ and *bla*_NDM_ groups. Both these gene families are homogenous with a low sequence variability between gene variants. It is therefore straightforward to design crRNAs that alone can recognize all known gene variants for each gene family. A combination of Cas9 loaded with these two RNAs can therefore be used to answer the clinically important question if carbapenem antibiotics can be used to treat the infection or not.

**Figure 5:**
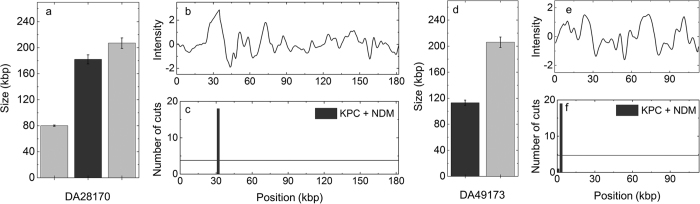
Detection of carbapenemase resistance genotype using a crRNA cocktail. (**a**) Sizes of all plasmids in isolate DA28170. The colors of the bars indicate if a plasmid is carrying the resistance gene (dark gray) or not (gray). (**b**) Consensus barcode of the middle sized plasmid in isolate DA28170. (**c**) Histogram showing the location of dsbreaks on the middle sized plasmid in isolate DA28170. (**d**) Sizes of all plasmids in isolate DA49173. The colors of the bars indicate if a plasmid is carrying the resistance gene (dark gray) or not (gray). (**e**) Consensus barcode of the small plasmid in isolate DA49173. (**f**) Histogram showing the location of dsbreaks on the small plasmid in isolate DA49173. The experiments were done in presence of Cas9 enzyme targeting both the *bla*_NDM_ and *bla*_KPC_ gene families. The horizontal lines in the histograms correspond to three standard deviations above the mean from the balls-in-boxes statistics. The results show that either a *bla*_KPC_ or a *bla*_NDM_ gene is present on the middle sized plasmid in DA28170 and the small plasmid in DA49173, and hence that both isolates show carbapenem resistance genotypes.

In Fig. 5, we study two carbapenem-resistant isolates known to carry a *bla*_KPC_ or a *bla*_NDM_ gene but with their genomes otherwise uncharacterized. In this case we used a mix of two crRNAs in the cleavage reaction, one that targets *bla*_KPC_ and one that targets *bla*_NDM_. In isolate DA28170 we detected three plasmids, 80, 182 and 207 kbp in size (Fig. 5a). For the 182 kbp plasmid we detected a distinct dsbreak, suggesting that this plasmid carries one of the carbapenemase genes. In the other two we did not observe any Cas9 cuts that were statistically significant (see Supplementary Information, Figure S4). Our assay was thus able to predict the phenotypic carbapenem resistance in this isolate and also demonstrate on which of the three plasmids the resistance gene is located.

The second isolate (DA49173) contains two plasmids, one 113 kbp in size and one 206 kbp in size (Fig. 5d–f and Supplementary Figure S5). For the 113 kb plasmid we detected one statistically significant Cas9-cut, but for the 206 kbp one we detected no Cas9-cut. Again, our Cas9 cocktail predicts the carbapenem resistance and demonstrates on which plasmid in the isolate the gene is located.

In the Supplementary Information (Figures S6 and S7) we show that by using Cas9 loaded with the *bla*_NDM_ and *bla*_KPC_ crRNAs, respectively, separately we can distinguish which cut that was caused by which Cas9/crRNA complex in Fig. 5 and we can thus potentially reveal more detailed information about the two isolates. That analysis reveals that our results agree with PCR in that DA28170 carries a *bla*_NDM_ gene and DA49173 carries a *bla*_KPC_ gene. In the Supplementary Information we also discuss the selection of crRNA for the *bla*_NDM_ gene family, where we in one case detected a false positive (Supplementary Information, Figures S8–S10).

## Discussion

We here demonstrate that it is possible to identify resistance genes in optical barcodes of individual bacterial plasmids by linearizing the circular plasmids with Cas9 loaded with a crRNA that recognizes the gene of interest. We do not run the assay to completion and can hence identify the plasmids in both their linear and circular forms. This is important since that for any linear barcode analyzed, the corresponding circular form has also been identified, which means that we can discard chromosomal DNA or plasmid fragments^16^. Also, if the plasmid contains more than one copy of the targeted gene, the assay will detect single cuts at two different positions along the barcode. If the gene copies are very close together, for example in direct repeats, the assay will not be able to detect the exact copy number but it will still detect the presence of the gene.

The assay has potential to be used as a rapid technique for detailed plasmid analysis that is applicable to low sample concentrations and that reveals as much information as possible in a single experiment. The assay measures the size of each plasmid, gives a fingerprint that can be used to identify and trace plasmids and it is now also possible to identify the presence of (resistance) genes of interest on the plasmids. This combined information obtained would require several different techniques and take up to one week to complete. The gene identification is an alternative to PCR, but where we also directly observe on which plasmid the gene is located if the sample contains more than one. One important advantage of this assay compared to PCR is that we obtain a detailed overview of the plasmid content in the sample even if the Cas9 does not cut any plasmid in the sample. For PCR, on the other hand, the assay will reveal no information about the plasmid content if the gene targeted is not present. One important further improvement compared to our earlier studies is that linear DNA pieces are analyzed together with circular pieces. This speeds up the data collection significantly since the plasmids do not have to be cut via illumination with light, a reaction that requires fine-tuning of the experimental conditions and therefore takes extra time. The full time from sample loading to data analysis is now down to approximately two hours, but can potentially be decreased even further.

For diagnostic use the assay is in its current format suitable for detecting a specific gene or group of genes of interest. As demonstrated for carbapenemases the assay supports multiplexing by adding Cas9 with several different crRNAs and will detect the presence of a gene belonging to one of the targeted groups. For the isolates in Fig. 5 we correctly predict the resistance since one plasmid in each isolate was cut by one of the Cas9/crRNA complexes. Since the assay is based on visualizing individual DNA molecules it has potential to be applied to low concentration samples without cultivation. This is important since it will decrease the time from sample collection to diagnosis and thereby enable rapid correct antibiotic treatment, minimizing the use of broad spectrum last resort antibiotics as well as antibiotics that are not effective. The assay could also be directly applicable to clinical samples containing uncultivable bacteria.

We analyze the *bla*_CTX-M_ family of genes in detail *in silico* to demonstrate the versatility of the assay. Since it is possible to design at least 100 different crRNAs for each gene, the selectivity for a gene, or group of genes, can be either very strict or very broad, depending on which crRNA that is selected. As a first example of such analysis we demonstrate that it is easy to discriminate the two most common groups of *bla*_CTX-M_ genes, group 1 and 9, since more than 99% of all crRNAs designed for one group will have at least three mismatches for any gene in the other. Within this context it is also important to address the specificity of the Cas9 enzyme. A recent report by Anderson *et al*. showed that 96% of the crRNAs with two mismatches were not functional^24^. For one mismatch the Cas9 can still work efficiently, in particular if the mismatch is far from the PAM-sequence. The specificity and off-target propensity of Cas9 is a field of intense research^27^,^28^ and we foresee that the assay can greatly benefit from this information to further optimize the selection of crRNAs with specific characteristics.

There are several possibilities of expanding the assay to further increase the reliability and resolution. From a clinical perspective, one critical feature is that it is important to identify a gene even if a point mutation has occurred. This can be accomplished by using several different crRNAs in the same reaction, targeting different regions of the same gene. It is then enough if one of these regions is intact in the sequence, the dsbreak will still occur and the gene will be identified. The compositions of these cocktails can be rationalized using bioinformatics tools to target the genes of interest and avoid false positives.

In the current format of the assay, one isolate is investigated at a time. To speed up the assay we foresee a future device that allows many samples to be investigated simultaneously. The software used here is already compatible with such a format and modern microscopes are easy to program to automatically collect images at several different locations on the device. We predict that devices where at least 10 samples are simultaneously analyzed are feasible in the near future. Such a device would allow either that many samples are simultaneously screened for a specific gene, that one sample is screened for several different genes, or a combination thereof. The time to do these kinds of experiments would not increase dramatically compared to the study here, since the enzymatic digestions can be run in parallel.

One important feature of the assay is that no additional labeling is needed to identify the gene of interest apart for the YOYO and netropsin used to form the barcodes. We are thus not limited by the low photon budget that comes with labeling via one or very few fluorophores that is used in most optical DNA mapping techniques^9^. This furthermore means that the assay could be directly transferable to more simple setups with a lower photon budget. One interesting example of such a setup is miniaturized fluorescence microscopes mounted on smartphone cameras. Wei *et al*. recently demonstrated that single DNA molecules can be visualized using such a microscope^29^. We foresee that, due to the large photon budget, our assay is directly transferable to such a format, either for DNA stretched in nanochannels or on glass, and should hence be applicable in laboratories around the world, including those in low-income countries.

Since it reveals detailed characteristics of plasmid content in a fast and simple fashion, we believe that our technique is the perfect complement to modern sequencing techniques. The assay gives a “birds-eye” view of the plasmid content in each sample and based on that samples of interest can, if needed, be selected for further analysis. It is also possible to do the experiments the other way around. By characterizing a key isolate using whole-genome sequencing, using e.g. the Pacific Biotechnology platform, we can predict a barcode for the plasmid(s) of interest and follow how they spread using the optical mapping assay. This would be of interest for example during resistance outbreaks.

To conclude, we have demonstrated that resistance genes can be directly identified in a fast and efficient way in single bacterial plasmids using Cas9 restriction. In the same experiment we reveal the number and size of all plasmids in an isolate, we show if a resistance gene or gene family is present and on which plasmid, and we also get a barcode that can be used for plasmid identification and tracing. The assay has applications from fundamental plasmid biology to epidemiology and clinical diagnostics, as demonstrated by the possibility to detect carbapenem resistant genotypes.

## Methods

### Bacterial strains and plasmids

Plasmid pUUH239.2 is a 220 kbp plasmid originally isolated from a clinical *K. pneumoniae* strain that caused a hospital outbreak in Uppsala, Sweden^21^. The plasmid used here was isolated from strain DA24337, an *E. coli* MG1655 derivative transconjugant with the plasmid. Plasmids pEC005A (67 kbp), pEC005B (139 kbp) and pEC019 (150 kbp) were originally isolated from *E. coli* from urinary tract infections as part of a screening of ESBL-producing *E. coli* in Sweden^20^. The plasmids were isolated from their respective original clinical *E. coli* strains DA25166 and DA25168. The plasmids containing *bla*_NDM_ or *bla*_KPC_ genes are previously uncharacterized apart from the presence of the respective carbapenemase gene. They were isolated from their respective original clinical *K. pneumoniae* isolates DA28170 and DA49173.

### Plasmid preparation

In order to extract plasmids the protocol for plasmid purification from the NucleoBond^®^ Xtra Midi kit (Macherey-Nagel) was used. For each isolate 100 ml of over night culture in low salt LB-medium (Sigma-Aldrich) was pelleted by centrifugation 5000 rcf, 10 minutes at 4 °C. The pellet was dissolved in Resuspension buffer, lysed and purified on columns according to the manufacturer’s recommendations. The eluted plasmid DNA was precipitated with isopropanol and washed once with 70% ethanol and dried at ambient temperature. The dried pellet was reconstituted in 50 μl TE-buffer. DNA concentration and purity was determined using Nanodrop.

### Sample preparation

The crRNAs were designed according to the following scheme. DNA sequences for all gene variants belonging to the beta-lactamase resistance gene families *bla*_CTX-M_, *bla*_KPC_ and *bla*_NDM_ were retrieved from http://www.lahey.org/studies/. Given a resistance gene variant (or replicon type), the set of potential crRNAs were identified by searching both its DNA strands for all possible PAM sequences (‘NGG’). Potential crRNAs were then derived by extracting the PAM sequence, together with the 20 upstream nucleotides. Next, the specificity of each potential crRNA was evaluated by calculating its similarity (proportion of matching nucleotides) to all gene variants within the gene family (see Supplementary Tables ST1 and ST2). The crRNAs used in this study were selected based on a high specificity to as many gene variants as possible. During the study different sequences of crRNA were used in order to target a specific gene of interest, see Supplementary Table ST3.

Both tracrRNA and crRNA were purchased from GE Healthcare and re-suspended in 10 mM RNase free Tris-HCl buffer (Sigma-Aldrich). gRNA was created by incubating 0.5 nmol tracrRNA with 0.5 nmol crRNA, in 1X NEBBuffer 3 (New England Biolabs) and 1X (0.1 μg/μL) BSA (New England Biolabs), for 30 minutes at 4 °C. Next, 10 μM (0.05 nmol) of gRNA was incubated with 600 ng of Cas9 (PNA Bio Inc.), 1X NEBBuffer 3 and 1X (0.1 μg/μL) BSA, at 37 °C for 15 minutes. Finally, 60 ng of DNA from the plasmid sample was added to the mixture followed by incubation at 37 °C for 1 hour.

Before adding the sample to the nanofluidic channels, the sequence specific pattern was created by staining the DNA at a molar ratio of typically 1:3.3 with YOYO-1 (YOYO, Invitrogen), and netropsin (Sigma Aldrich), ratio 100:1 with respect to YOYO. λ-DNA (48502 bp, New England Biolabs) was included in the sample as an internal size reference. First, samples were mixed in 0.5X TBE (Tris-Borate-EDTA, Medicago, diluted with MQ from 10X tablets) and incubated at 50 °C for 30 minutes. Samples were then diluted with MQ in order to reach a final buffer concentration of 0.05X and 0.2 μM (bp) DNA (0.1 μM Cas9 treated plasmid DNA + 0.1 μM λ-DNA). Photonicking was supressed by adding β-mercaptoethanol (BME, Sigma-Aldrich) at 2% (v/v).

### Experimental procedure

Nanofluidic chips in fused silica were fabricated using standard methods as described elsewhere^11^. Channels with dimensions of 100 × 150 nm^2^, and a length of 500 μm were used in order to stretch the DNA molecules. In total four loading wells in each chip were etched and connected two by two with microchannels, which in turn are spanned by the nanochannels. In order to achieve uniform conditions, the channels were pre-wetted with 0.05X TBE buffer and 2% v/v BME. A sample volume of 10 μL was loaded into the chip and DNA molecules were forced in to the nanofluidic channels using pressure driven flow of nitrogen gas. Using an inverted microscope (Zeiss AxioObserver.Z1) with a 100x oil immersion objective (Zeiss, NA = 1.46), both circular and linear DNA molecules were imaged with an EMCCD camera (Photometrix Evolve). In total a series of up to 200 images with an exposure time of 100 ms were obtained from each DNA molecule in order to measure its size (circular and linear) and obtain the sequence specific barcode (linear).

### Data analysis and statistical analysis

The purpose of the data and statistical analyses are to: (i) for the case of samples with more than one type of plasmid, cluster DNA molecules based on plasmid size (ii) for a given cluster, align the associated DNA barcodes using maximum Pearson correlation coefficients for each potential alignment (where each alignment is characterized by a direction and cyclical shift) and average the aligned barcodes to create and average the aligned barcodes to create so called consensus barcodes. In this step outliers, such as chromosomal fragments or plasmids that have been broken more than once or are poorly stained, are also removed. (iii) Based on the alignment from (ii), detect whether the dsbreaks occurred at random positions (gene not present), or whether all dsbreaks occurred at the same position (gene present). The details are presented below.

The first step in our data analysis procedure is to cluster DNA barcodes according to size. Using the extension of imaged λ-DNA molecules, a reference value of number of basepairs per pixel for each separate experiment was obtained, allowing for plasmid sizing. Intact plasmids are circular by nature, making it possible to separate linear fragments from circular when extended in the nanochannels due to the higher intensity of emitted light from the double folded plasmids^19^. Plasmids in their native circular form, which will be present even if the plasmid is targeted with Cas9 since the reaction is not run to completion, were used to calculate the sizes of plasmids present in the isolates. To convert the sizes of circular plasmids to the linear form we use a previously reported^14^,^19^ conversion factor of 1.8. For each detected plasmid size all linear DNA fragments +/− 20% in size were then merged into a cluster.

In the second step we used DNA barcodes within a given cluster to generate a consensus barcode of the plasmid^15^. The consensus barcode consists of the average of the individual barcodes that pass a certain cross correlation threshold (typically 0.65, Supporting Figure S11) once aligned to one another. The threshold was set as low as possible in order to maximize the amount of data used, but still high enough to separate the correct plasmid barcodes from chromosomal DNA and fragmented plasmid DNA that might be present in the sample. Doing this, the resulting consensus barcode represents the underlying sequence well and the percentage of cuts in the most filled bin is not lower compared to a higher cutoff where fewer barcodes can be used for the cut statistics. Using a correlation coefficient threshold also makes it possible to detect and separate plasmids of the same size based on their underlying sequence.

In the third and final step, we detect whether the dsbreak occurred at random position (gene not present), or whether a majority of the dsbreaks occurred at the same position (gene present). To that end, the information obtained when generating the consensus barcode is also used to detect the presence of the Cas9 targeted sequence on a plasmid. Since a circular plasmid can be broken at any position along the DNA sequence, one needs to consider each possible start position, as well as its mirror images, when merging two individual plasmid barcodes during consensus generation (Fig. 1a). Besides a Cas9-targeted dsbreak in the plasmid, a dsbreak can be induced in the plasmid by either light exposure, or due to mechanical forces during plasmid extraction or pipetting. This means that when studying the position of the dsbreaks of each individual plasmid in the consensus, there will almost always be some breaks that are not aligned, i.e. broken at the same position. However, if a plasmid in the isolate has the complementary sequence to the crRNA, the majority of the plasmids will be linearized at the same position, and hence the presence of the targeted sequence can be detected. In order to determine how large the fraction of dsbreaks that are aligned need to be to tell if a gene is present or not, a simple “balls-in-boxes” approach was applied (see Supplementary Methods for details) – in short, if the observed number of dsbreaks in the bin (see below) with the most number of dsbreaks is more than three standard deviations away from the expected value, we deem the number of dsbreaks “significant”. In bullet point form our gene-ID method proceed as follows:

- Treat each pixel as a “box”. A dsbreak event corresponds to a ball positioned in a given box. There are n boxes (pixels) and r balls. For a given cluster (point (i)), the number of balls, r, is the number of kymographs that pass the cross correlation threshold.
- Randomly distribute the r balls into the n boxes. Bin the data into bins of size = four (overlapping) boxes in the way that optimizes the amount of balls that can be found in one bin, and count the number of balls in the most-filled bin. Repeat this 10000 times, and calculate mean number of balls in the most-filled bin and the associated standard deviation. If the observed number of dsbreaks in the most-filled bin is larger than the mean + 3 standard deviations, the result is deemed significant and we declare the gene to be present.
- In order to detect more than one gene on the same plasmid, the process is first applied to the most-filled bin as described above. In order to detect the second gene, we “remove” the most filled bin (and the corresponding balls) from the simulations, and count the number of events in the second most-filled bin based on 10000 random realizations. From this data we again calculate the mean and standard deviation. If the measured number of events in the second most-filled bin is larger than this new mean plus three standard deviations, the second most-filled box is also deemed to have a significant number of events and a second gene is also said to be present. We repeat this procedure (for the third most-filled bin etc) until we get no more bins that pass the threshold.

The rationale for using bins of size four pixels is that the standard deviation of optical point spread function is approximately 300 nm, which means that four pixels corresponds to two units of the width of the point spread function (the point spread function defines the resolution of the optical maps).

## Additional Information

**How to cite this article**: Müller, V. *et al*. Direct identification of antibiotic resistance genes on single plasmid molecules using CRISPR/Cas9 in combination with optical DNA mapping. *Sci. Rep.* **6**, 37938; doi: 10.1038/srep37938 (2016).

**Publisher's note:** Springer Nature remains neutral with regard to jurisdictional claims in published maps and institutional affiliations.

## Supplementary Material

Supplementary Information
