# Supplementary material for: Direct identification of antibiotic resistance genes on single plasmid molecules using CRISPR/Cas9 in combination with optical DNA mapping
Source: Sci Rep. 2016 Dec 1;6:37938. doi: 10.1038/srep37938 (PMC5131345; doi:10.1038/srep37938)
Supplement: Supplementary Information [file srep37938-s1.pdf]

Supplementary: Direct identification of antibiotic resistance genes on single plasmid molecules using CRISPR-Cas9 in combination with optical DNA mapping

Vilhelm Müller[1], Fredrika Rajer[2], Karolin Frykholm[1], Lena K. Nyberg[1], Saair Quaderi[1,3], Joachim Fritzsche[4], Erik Kristiansson[5,6], Tobias Ambjörnsson[3], Linus Sandegren[2] and Fredrik Westerlund[1]

[1]Department of Biology and Biological Engineering, Chalmers University of Technology, Gothenburg, Sweden
[2]Department of Medical Biochemistry and Microbiology, Uppsala University, Uppsala, Sweden
[3]Department of Astronomy and Theoretical Physics, Lund University, Lund, Sweden
[4]Department of Applied Physics, Chalmers University of Technology, Gothenburg, Sweden
[5]Department of Mathematical Sciences, Chalmers University of Technology/University of Gothenburg, Gothenburg, Sweden
[6]Centre for Antibiotic Resistance Research (CARe), University of Gothenburg, Gothenburg, Sweden

[17 pages, 11 figures, 3 tables]

S.M Supplementary Methods

The output from the experimental assay presented in the main text is in the form of r DNA barcodes each consisting of n pixels. For each barcode one of the pixels contains a cut. The statistical challenge is to determine whether, out of the M cut positions, a “sufficient” number of cuts ended up in the same bin (consecutive set of pixels). This problem is the same as that of throwing r balls into n boxes. In the rest of this Supplementary we investigate the associated balls-in-boxes statistics.

S.M.1 Balls-in-boxes statistics

Consider r balls which are to be placed in n boxes. For each new ball, a box is picked with uniform probability (i.e. each of the boxes is equally probable to receive a ball). A box can contain an arbitrary number of balls. The statistics of such a system has been investigated before[2, 3], however not with respect to the observable (see below) used herein.

In this study we are interested in the statistics for the maximum of the number of balls in a given bin i; a bin is defined to be a set of D consecutive boxes. Mathematically, we thus study the random number X_i, which is simply the number of balls in the consecutive boxes $\{i, i+1, \ldots, i+D-1\}$ (bin i) and defined as

$$X_i = r_i + r_{i+1} + \ldots r_{i+D-1}, \tag{S.1}$$

where r_i is the number of balls in box i $(i = 1, 2, ..., N)$. For circular symmetric systems, as relevant for circular DNA as considered herein, we use $r_{i+N} = r_i$. Note that the bins, by construction, are overlapping (bin 1 contains boxes $\{1, 2\ldots, D\}$, bin 2 contains boxes $\{2, 3, \ldots, D+1\}$ etc). The reason for using overlapping bins is to avoid any arbitrariness regarding choice of “start box” for the circular DNA considered herein. Due to the overlap, the random numbers X_i are, by construction, correlated (for $D > 1$). We now seek the probability density, $\rho(\hat{X}|r, n)$ for the maximum $\hat{X} = \max\{X_1, X_2, \ldots X_N\}$, i.e. for *the probability that the D-sized bin with the largest number of balls had $\hat{X}$ balls in it.*

For the statistics we use a simple approach in the main text: if the observed number of balls $\hat{X}_{\text{observed}}$ in the bin with most balls is 3 standard deviations away from the expected value, then the observed value is deemed “significant” (gene is present). The analysis thus requires the following quantities: the mean number of balls in the “most-filled” bin,

$$\langle \hat{X} \rangle = \sum_{\hat{X}=0}^{r} \hat{X} \rho(\hat{X}|r, n) \tag{S.2}$$

and the associated standard deviation, σ, where $\sigma^2 = \langle \hat{X}^2 \rangle - \langle \hat{X} \rangle^2$, where

$$\langle \hat{X}^2 \rangle = \sum_{\hat{X}=0}^{r} \hat{X}^2 \rho(\hat{X}|r, n) \tag{S.3}$$

Evaluating $\langle \hat{X} \rangle$ and σ analytically for arbitrary choice of D is a challenging task (since the random numbers X_i are correlated), see Section S.M.2. Instead, we here revert to simulations: r balls were stochastically "thrown" into n boxes.[1] For each such simulation run, we calculated the X_i values using Eq. (S.1), and subsequently the maximum, $\hat{X} = \max\{X_1, X_2, \ldots X_N\}$, was

[1] A uniform random R, number was obtained and converted into a box label n_{random}, i.e., a uniform random number in the range $[1, n]$, according to $n_{\text{random}} = [nR] + 1$, where $[Z]$ denotes truncation to the closest lower integer of the real valued number Z.

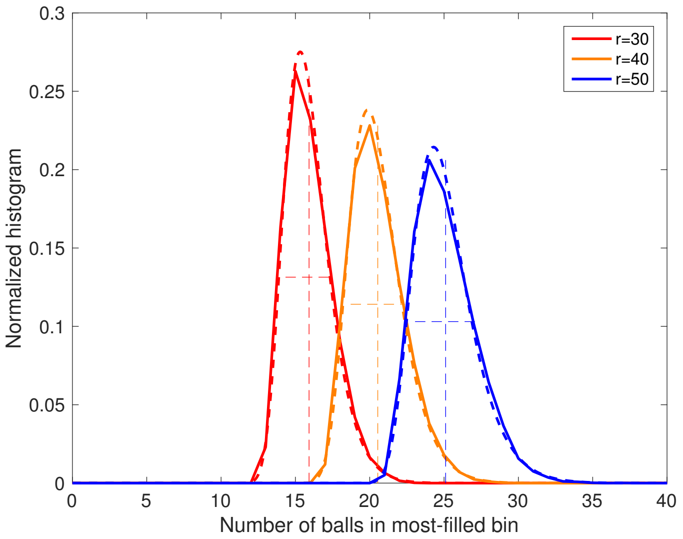

Figure S1: **Histogram for number of balls in the "most-filled" bin.** r number of balls were stochastically thrown into n boxes using a procedure as described in Sec. S.M.1. Parameters: number of balls $r = 30, 40, 50$, number of boxes $n = 10$, bin size $= D = 4$, and number of simulations, $N_{\text{sim}} = 10^4$. The estimated sample means (standard deviation) are indicated as vertical (horizontal) lines. Dashed curves are Gumbel PDFs fitted to the data using the maximum likelihood method (function `fitdist` in Matlab).

stored for that run. This process was then repeated over N_{sim} simulation runs. From the data, the sample mean and associated sample estimate for the standard deviation were computed as estimators for $\langle \hat{X} \rangle$ and σ, respectively. Figure S1 shows a histogram of the $\hat{X}$ values for a few different r (number of balls).

Assuming that the functional form of $\rho(\hat{X}|r, n)$ is known, our choice of threshold (mean + three standard deviation) for deeming observed values significant can be translated into a p-value threshold, p_{thresh}, according to

$$p_{\text{thresh}} = \int_{\langle \hat{X} \rangle + Z\sigma}^{\infty} \rho(\hat{X}|r, n) d\hat{X}, \tag{S.4}$$

where, here, $Z = 3$. In Fig. S1 we fitted to Gumbel PDFs which appear to have a functional form consistent with the simulated histograms (the Gumbel distribution is the limiting distribution for the largest element in a set of independent random numbers [1]). Using the cumulative distribution for the Gumbel PDF, and its known mean and variance, one straightforwardly shows that

$$p_{\text{thresh}} = 1 - \exp[-\exp(-(Z\pi/\sqrt{6} + \gamma))], \tag{S.5}$$

where γ is the Euler-Mascheroni constant ($= 0.5772$). Setting $Z = 3$ above, we find $p_{\text{thresh}} = 1 - 0.988 = 1.2\%$. The quantity $1 - p_{\text{thresh}} = 98.8\%$ gives the proportion of all events which "should" end up in the most-filled bin if the "zero model" (balls are uniformly distributed among boxes) is correct.

S.M.2 Analytic results are difficult to obtain for $D > 1$

In this section, we show that obtaining analytical results for the quantity $\rho(\hat{X}|r,n)$, see Sec. S.M.1, is a difficult task if $D > 1$.

Consider again r balls which are to be placed in n boxes. The joint probability that boxes j ($j = 1, ..., n$) contains r_j balls is the multinomial distribution[2, 3]

$$P(r_1, r_2, ..., r_n) = \frac{r!}{r_1! r_2! \cdots r_n!} p_1^{r_1} p_2^{r_2} \cdots p_r^{r_n} \tag{S.6}$$

and

$$r = r_1 + r_2 + ... + r_n \tag{S.7}$$

where p_j is the probability for box j ($p_1 + p_2 + \ldots p_n = 1$). We here assumed that a ball is equally likely to be placed in any of the boxes, i.e.,

$$p_1 = p_2 = \ldots = p_n = \frac{1}{n}. \tag{S.8}$$

Based on the multivariate distribution above, any observable can, in principle be calculated analytically. In practice, calculating such observables may not be straightforward, however.

In order to obtain a formal expression for the quantity of interest here, $\rho(\hat{X}|r,n)$, we introduce $Q(\hat{X}|r,n)$, which is the probability that *no bin has more than $\hat{X}$ balls*. Formally,

$$Q(\hat{X}|r,n) = \sum_{X_1 \leq \hat{X}, X_2 \leq \hat{X}, ..., X_n \leq \hat{X}} \phi(X_1, X_2, ..., X_n) \tag{S.9}$$

where

$$\phi(X_1, X_2, \ldots X_N) = \sum_{r_1, r_2, \ldots r_N} \prod_{i=1}^{N} \delta_{X_i, r_i + r_{i+1} + \ldots r_{i+D-1}} P(r_1, r_2, \ldots r_N) \tag{S.10}$$

is the joint probability density for $\{X_1, X_2, \ldots X_N\}$ and $\delta_{n,m}$ is the Kronecker delta function, i.e., $\delta_{n,m} = 1$ if $n = m$ and $= 0$ otherwise. The exact expression for the multinomial $P(r_1, r_2, \ldots r_N)$ is given in Eq. (S.6). The quantity $Q(\hat{X}|r,n)$ is related to $\rho(\hat{X}|r,n)$ according to

$$Q(\hat{X}|r,n) = \sum_{\hat{X}'=0}^{\hat{X}} \rho(\hat{X}'|r,n) \tag{S.11}$$

i.e. the probability that all boxes has $\leq \hat{X}$ balls in them is the sum of probabilities that the largest number of balls in any box is $0, 1, 2, ..., \hat{X}$. "Inverting" the expression above we have

$$\rho(\hat{X}|r,n) = Q(\hat{X}|r,n) - Q(\hat{X} - 1|r,n) \tag{S.12}$$

for $\hat{X} \geq 1$ and completed by $\rho(0|r,n) = Q(0|r,n)$. It is reassuring that the $\rho(\hat{X})$ above is normalized: $\sum_{\hat{X}=0}^{\infty} \rho(\hat{X}|r,n) = Q(\infty|r,n) = 1$.

The analysis above is formally exact. However, in practice analytically evaluting the multi-dimensional sum in the expression for $Q(\hat{X}|r,n)$, see Eq. (S.9), and calculate associated observables, is a complicated task. For the case $D = 1$ it was shown by Ewens and Wilf [2] that calculating $Q(\hat{X}|r,n)$ can be done exactly using a recursion relation. It was later argued in [3] that, for most practical purposes in statistics, a Poisson approximation of the multinomial above provides similar results, for $D = 1$. Extending these previous results to the case of interest here, namely $D \geq 2$, is complicated by the fact that the X_is are correlated. We therefore leave this problem as a future challenge.

S.F Supplementary Figures

S.F.1 Size Comparisons

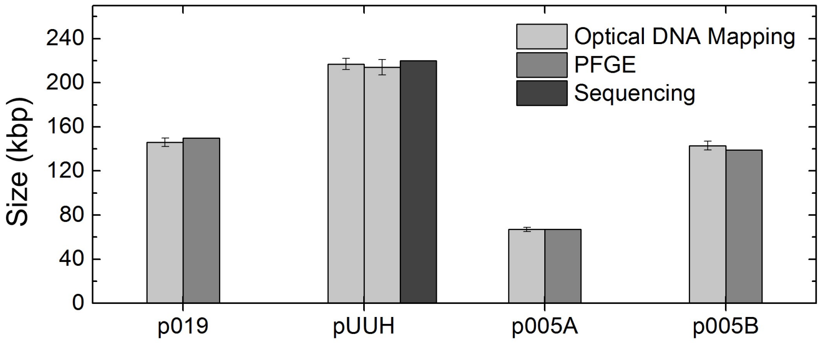

Figure S2: **Plasmid sizes.** Sizes of the plasmids studied in Figure 2 and 4 obtained from optical mapping in this study (light gray) compared to PFGE (gray) and sequencing (dark gray) showing excellent similarity. The two light gray bars for pUUH239.2 correspond to plasmids cut by Cas9 targeting the $bla_{CTX\text{-}M\text{-}15}$ gene (first) and the REPA2 gene (second), respectively, demonstrating the reproducibility of the method.

S.F.2 Consensus Barcodes for pUUH239.2

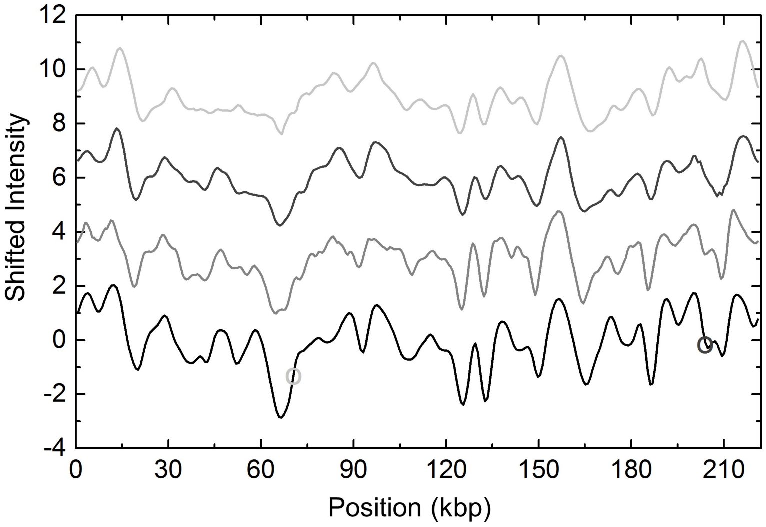

Figure S3: **Consensus barcodes for pUUH239.2.** Consensus barcodes for plasmid pUUH239.2 cut with crRNA targeting RepA2 (light gray), $bla_{CTX\text{-}M\text{-}15}$ (dark gray), and light (gray) compared to the theoretical barcode (black) showing excellent overlap. The circles point out the location of the RepA2 gene (light gray) and the CTXM-15 gene (dark gray).

S.F.3 Plasmids (80 kb + 207 kb) in DA28170 not cut by Cas9 with crRNA-cocktail

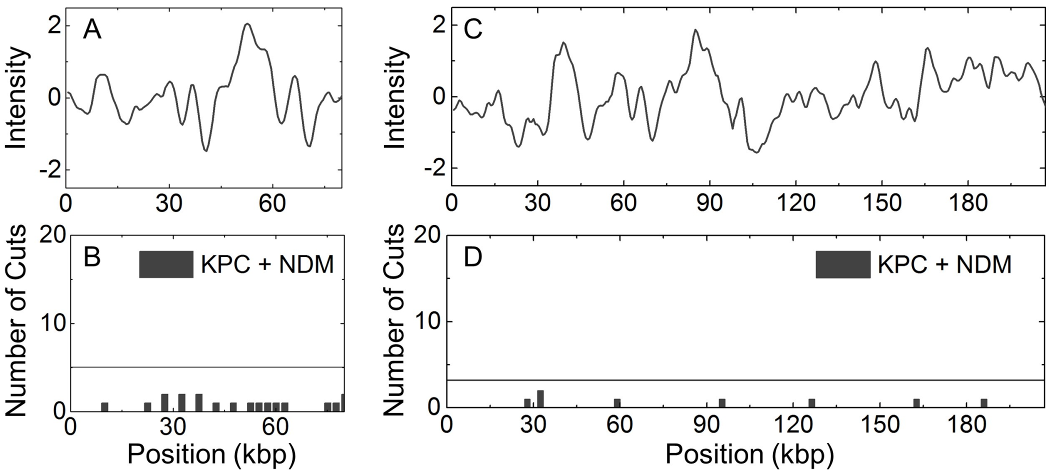

Figure S4: **Plasmids in isolate DA28170 not cut by Cas9** A) Consensus barcode for the small plasmid (80 kbp) in sample DA28170. B) Histogram showing the location of dsbreaks on the small plasmid in isolate DA28170. C) Consensus barcode for the large plasmid (207 kbp) in sample DA28170. D) Histogram showing the location of dsbreaks on the large plasmid in isolate DA28170. The sample has been exposed to Cas9 targeting the bla_{NDM} and bla_{KPC} gene families. The horizontal lines in the histograms correspond to three standard deviations above the mean from the balls in boxes statistics.

The small (80 kbp) and large (207 kbp) plasmids in isolate DA28170 are not cut by the cocktail containing Cas9 targeting the bla_{NDM} and bla_{KPC} gene families showing that neither of these gene families are present on any of these plasmids.

S.F.4 Plasmid (206 kbp) in DA49173 not cut by Cas9 with crRNA-cocktail

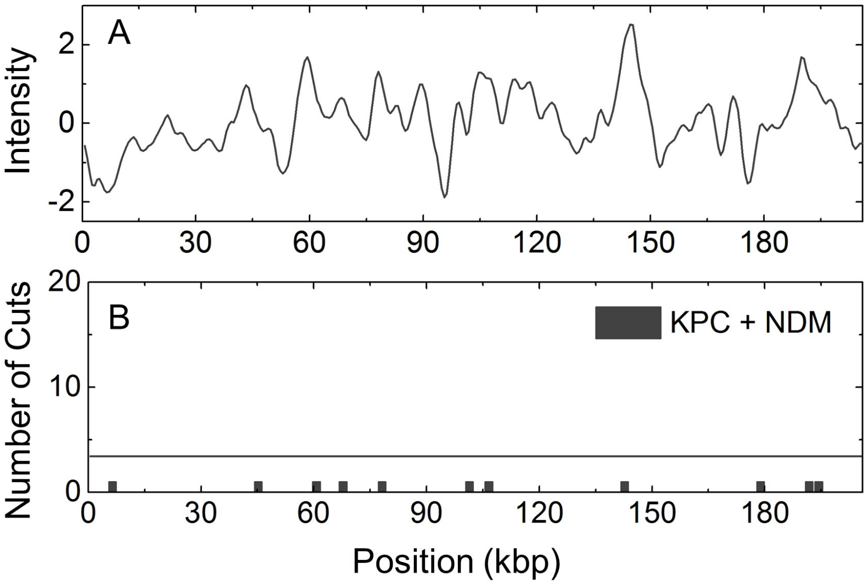

Figure S5: **Plasmid in isolate DA49173 not cut by Cas9 with crRNA-cocktail** A) Consensus barcode for the large plasmid (206 kbp) in sample DA49173. B) Histogram showing the location of dsbreaks on the large plasmid in isolate DA49173. The sample has been exposed to Cas9 targeting the bla_{NDM} and bla_{KPC} gene families. The horizontal line in the histogram correspond to three standard deviations above the mean from the balls in boxes statistics.

The large plasmid (206 kbp) in isolate DA49173 is not cut by the cocktail containing Cas9 targeting the bla_{NDM} and bla_{KPC} gene families showing that neither of these gene families are present on this plasmid.

S.F.5 Cocktail and bla_{NDM} Control for DA28170

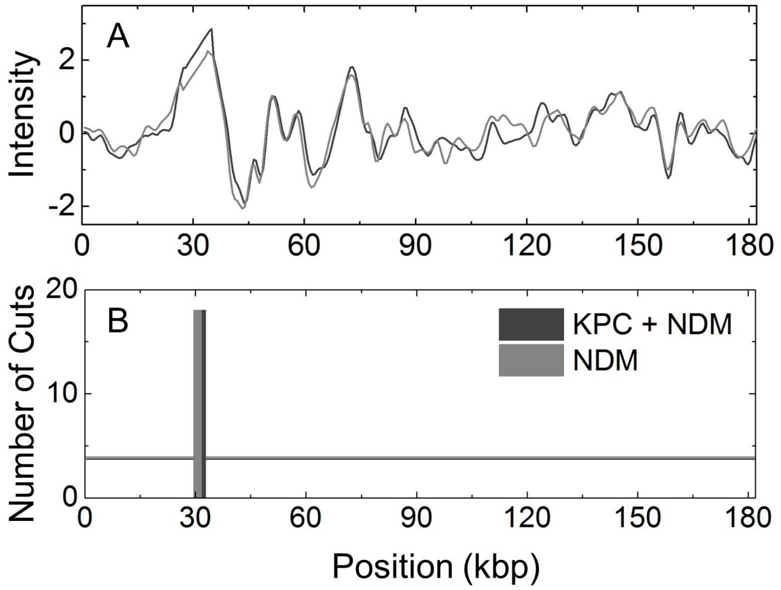

Figure S6: **Cas9 cut on middle sized plasmid in isolate DA28170** A) Consensus barcode for the middle sized plasmid in sample DA28170. B) Histogram showing the location of dsbreaks on the middle sized plasmid in isolate DA28170. The sample has been exposed to either the cocktail targeting the bla_{KPC} and bla_{NDM} gene families (dark gray) or Cas9 targeting the bla_{NDM} gene family only (gray). The horizontal lines in the histogram correspond to three standard deviations above the mean from the balls in boxes statistics.

Using a Cas9 that only targets the bla_{NDM} gene family on isolate DA28170 we demonstrate that the gene that causes the carbapenem resistance belongs to this group and is located on the middle sized plasmid. The cut occurs at exactly the same location when using the cocktail or the bla_{NDM} crRNA only.

S.F.6 Cocktail and bla_{KPC} control for DA49173

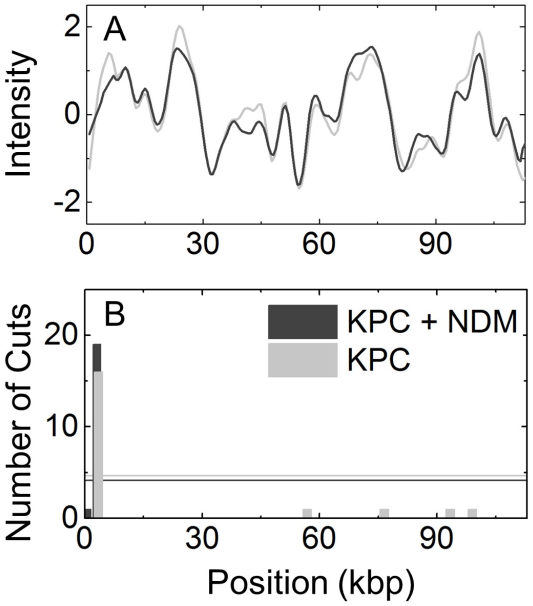

Figure S7: **Cas9 cut on small plasmid in isolate DA49173** A) Consensus barcode for the small plasmid in sample DA49173. B) Histogram showing the location of dsbreaks on the small plasmid in isolate DA49173. The sample has been exposed to either the cocktail targeting the bla_{KPC} and bla_{NDM} gene families (dark gray) or Cas9 targeting the bla_{KPC} gene family only (light gray). The horizontal lines in the histograms correspond to three standard deviations above the mean from the balls in boxes statistics.

Using a Cas9 that only targets the bla_{KPC} gene family on isolate DA49173 we demonstrate that the gene that causes the carbapenem resistance belongs to this group and is located on the small plasmid. The cut occurs at exactly the same location when using the cocktail or the bla_{KPC} crRNA only.

S.F.7 Cocktail with crRNA bla_{NDM}*(2)*

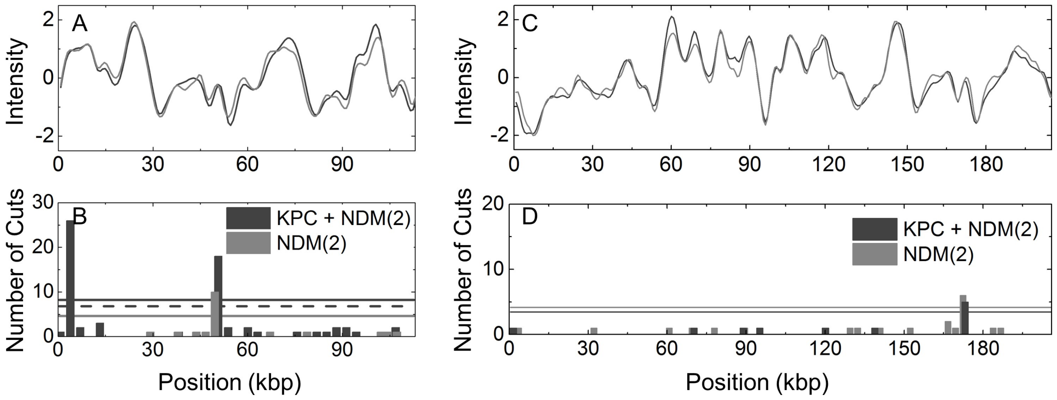

Figure S8: **DA49173 cut with cocktail containing bla_{NDM}*(2)*** A) Consensus barcodes for the small plasmid in DA49173. B) Histogram showing the location of dsbreaks on the small plasmid in isolate DA49173. C) Consensus barcodes for the large plasmid in DA49173. D) Histogram showing the location of dsbreaks on the large plasmid in isolate DA49173. The sample has been exposed to either a cocktail containing Cas9 loaded with a crRNA that targets the bla_{KPC} gene family and the bla_{NDM}*(2)* crRNA (dark gray) or the bla_{NDM}*(2)* crRNA only (gray). The horizontal lines in the histograms correspond to three standard deviations above the mean from the balls in boxes statistics. The dashed line (dark grey) in B) indicates the threshold value for the second cut that was obtained on the small plasmid when using the crRNA cocktail.

During our initial studies of isolate DA49173 with Cas9 targeting the bla_{NDM} gene family (using a crRNA we term bla_{NDM}*(2)*) together with Cas9 targeting the bla_{KPC} gene family we noticed two statistically significant cuts in the small plasmid and one in the large plasmid. Two of these three cuts remained when we used the bla_{NDM}*(2)* crRNA only, even though PCR was negative for bla_{NDM}, suggesting that bla_{NDM}*(2)* cuts the plasmid also when the bla_{NDM} gene is not present. Changing to the bla_{NDM} crRNA used in the main text, both these cuts disappeared, confirming that they were false positives caused by bla_{NDM}*(2)*.

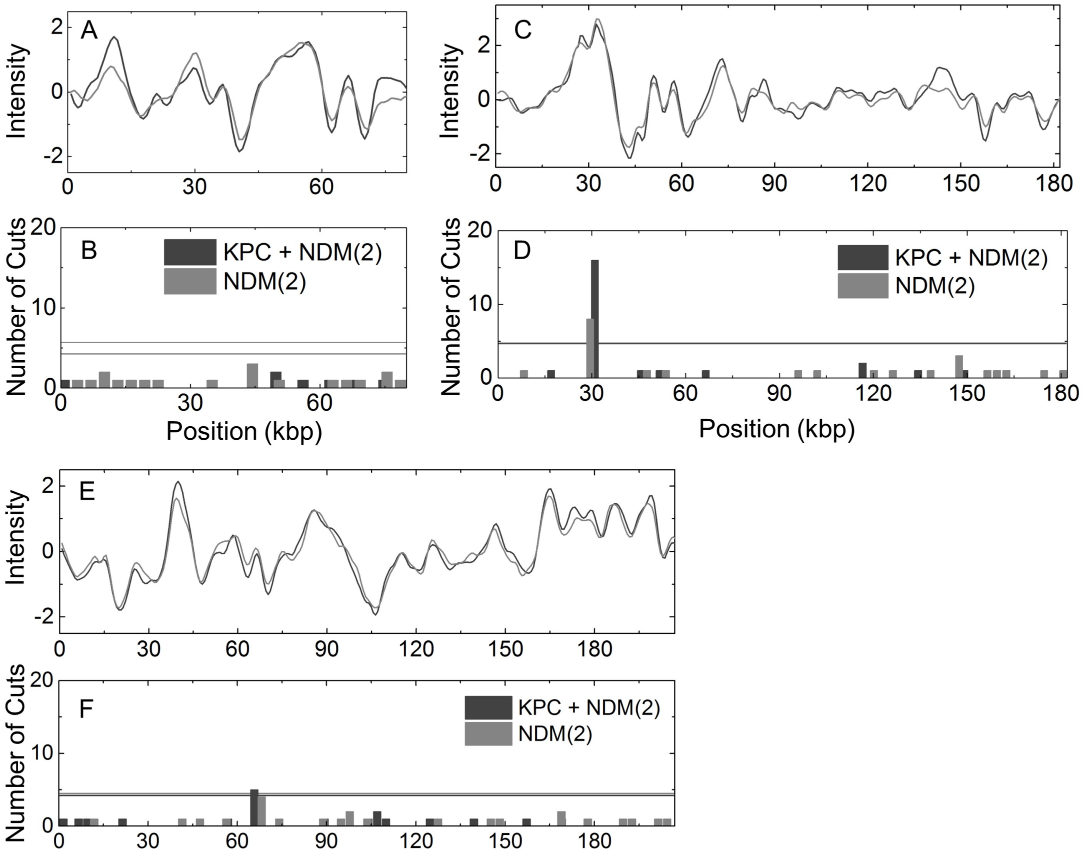

Figure S9: **DA28170 cut with cocktail containing *bla*$_{NDM}$*(2)*** A) Consensus barcodes for the small plasmid in DA28170. B) Histogram showing the location of dsbreaks on the small plasmid in isolate DA28170. C) Consensus barcodes for the middle sized plasmid in DA49173. D) Histogram showing the location of dsbreaks on the middle sized plasmid in isolate DA49173. E) Consensus barcodes for the large plasmid in DA49173. F) Histogram showing the location of dsbreaks on the large plasmid in isolate DA49173. The sample has been exposed to either a cocktail containing Cas9 loaded with a crRNA that targets the bla_{KPC} gene family and the bla_{NDM}*(2)* crRNA (dark gray) or the bla_{NDM}*(2)* crRNA only (gray). The horizontal lines in the histograms correspond to three standard deviations above the mean from the balls in boxes statistics.

We then returned to DA28170 and did the analysis with the new crRNA for the bla_{NDM} gene family also on this isolate. Doing this we realized that the bla_{NDM}*(2)* caused a false positive also on the large plasmid in DA28170 while the cut on the middle sized plasmid was there for both crRNAs.

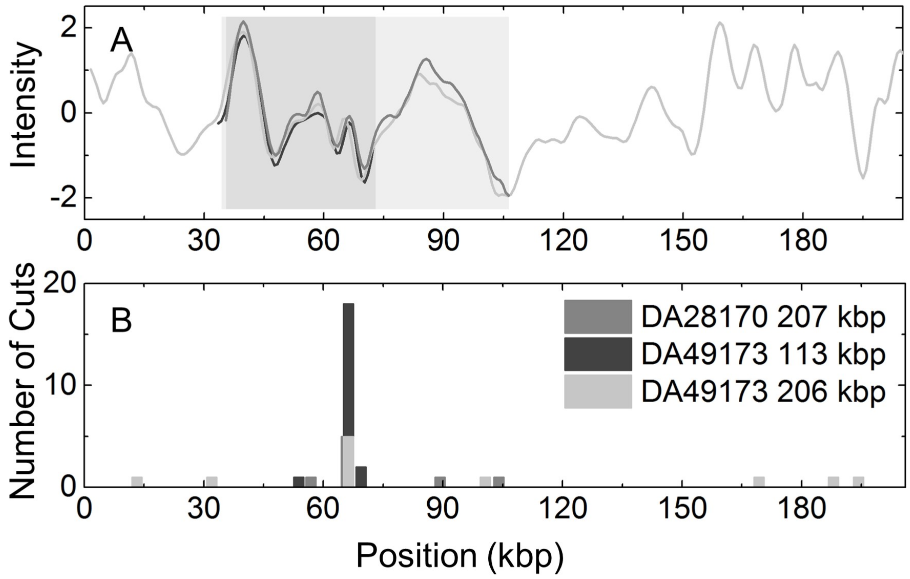

Figure S10: **Common region in plasmids from DA28170 and DA49173** A) Consensus barcode of the large plasmid (206 kbp) in sample DA49173 (light gray). The light gray shading indicates a region with excellent overlap between the large plasmid in DA49173 and the large plasmid (207 kbp) in DA28170 (gray). The dark gray shading indicates a region with excellent overlap between the large plasmid in DA49173 and the small plasmid (113 kbp) in DA49173 (dark gray). B) Histogram showing the location of dsbreaks on the three plasmids when the samples have been exposed to Cas9 loaded with the $bla_{NDM}(2)$ crRNA. The horizontal lines in the histograms correspond to three standard deviations above the mean from the balls in boxes statistics.

During our analysis of the false positives using Cas9 loaded with the $bla_{NDM}(2)$ crRNA we noticed that the dsbreaks in all three plasmids occur at a location in the barcodes that are very similar for the three plasmids. Comparing the large plasmid in DA49173 and the large plasmid in DA28170, this region is as large as around 70 kbp while when comparing the large and small plasmids in DA49173 the region is around 40 kbp. The Cas9 cuts the plasmids at the exact same location in all three barcodes suggesting that all three plasmids have a common region and that the false positive is caused by the same sequence in all three plasmids and hence that it is the same false positive for all three plasmids. We do not know what the sequence is, if it corresponds exactly to the crRNA used or if has a slight variation in sequence.

S.F.8 Threshold value for cross correlation

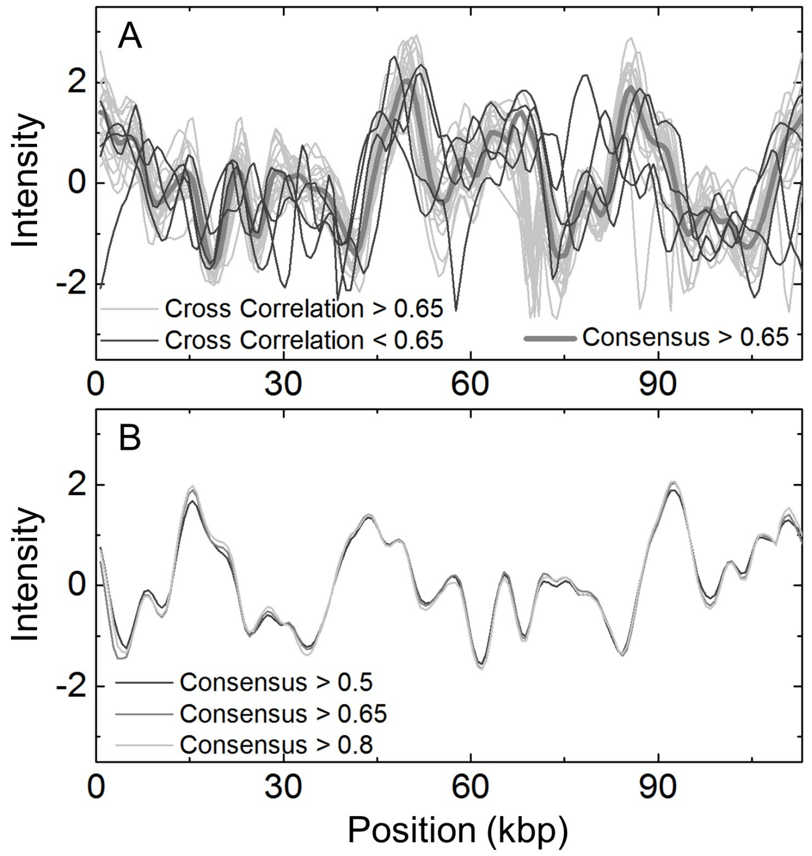

Figure S11: **Effect of cross correlation threshold value** A) All individual barcodes from DNA molecules with a size of 113 kbp +/- 20% found in DA49173 when using Cas9 loaded with crRNA targeting the KPC resistance gene. Individual barcodes with cross correlation values higher than 0.65 when generating the consensus barcode (light gray) and individual barcodes with cross correlation values lower than 0.65 (dark gray). The corresponding consensus barcode used in later processing steps for both gene detection as well as plasmid ID is shown in gray (bold). B) Consensus barcodes generated from individual barcodes in A) using three different cross correlation thresholds.

Using a cross correlation threshold of 0.65 barcodes that do not represent the entire plasmid sequence, such as chromosomal DNA or fragmented plasmid DNA, but still are of the same size will be sorted out (black lines). This is important since including non plasmid barcodes will reduce the sensitivity of gene detection in subsequent data processing steps. The resulting consensus barcodes generated from the individual barcodes that pass the threshold value do not change dramatically when the cross correlation threshold is altered. This shows that a threshold value of 0.65 is high enough to maintain good quality of the data, but still not so high that to much data will be excluded.

S.T Supplementary Tables

S.T.1 $bla_{CTX\text{-}M\text{-}14}$ crRNA sequences used for in silico analysis of the $bla_{CTX\text{-}M}$ gene family

Sequence on sense strand (5' to 3')	Start	Hits[a]	Sequence on antisense strand (5' to 3')	Start	Hits[a]
TGGTGACAAAGAGAGTGCAACGG	2	49	CCGCTGCTGCTGGGCAGCGCGCC	52	48
AGTGCAACGGATGATGTTCGCGG	15	47	CCGCTTTATGCGCAGACGAGTGC	73	46
GCAACGGATGATGTTCGCGGCGG	18	47	CCGCAGATAATACGCAGGTGCTT	164	48
ACGGATGATGTTCGCGGCGGCGG	21	47	CCAATGTGCAGTACCAGTAAAGT	208	51
GGCGTGCATTCCGCTGCTGCTGG	42	49	CCAGTAAAGTTATGGCGGCCGCG	221	43
GCGTGCATTCCGCTGCTGCTGGG	43	49	CCGCGGCGGTGCTTAAGCAGAGT	239	41
GCTTTATGCGCAGACGAGTGCGG	75	47	CCTGTCGAGATCAAGCCTGCCGA	289	50
GAGTGCGGTGCAGCAAAAGCTGG	90	47	CCTGCCGATCTGGTTAACTACAA	304	50
TGCGGTGCAGCAAAAGCTGGCGG	93	48	CCGATCTGGTTAACTACAATCCG	308	50
GCAGCAAAAGCTGGCGGCGCTGG	99	49	CCGATTGCCGAAAAACACGTCAA	328	49
GCGGCGCTGGAGAAAAGCAGCGG	112	62	CCGAAAAACACGTCAACGGCACA	335	50
GCGCTGGAGAAAAGCAGCGGAGG	115	47	CCGCGTTGCAGTACAGCGACAAT	383	51
CGCTGGAGAAAAGCAGCGGAGGG	116	47	CCGCCATGAACAAATTGATTGCC	407	53
TGGAGAAAAGCAGCGGAGGGCGG	119	47	CCATGAACAAATTGATTGCCCAG	410	53
GAAAAGCAGCGGAGGGCGGCTGG	123	47	CCCAGCTCGGTGGCCCGGGAGGC	428	51
AAAAGCAGCGGAGGGCGGCTGGG	124	47	CCAGCTCGGTGGCCCGGGAGGCG	429	51
CGATACCGCAGATAATACGCAGG	159	48	CCCGGGAGGCGTGACGGCTTTTG	441	51
AATACGCAGGTGCTTTATCGCGG	172	49	CCGGGAGGCGTGACGGCTTTTGC	442	51
GTGCAGTACCAGTAAAGTTATGG	213	50	CCCGCGCGATCGGCGATGAGACG	464	51
CAGTACCAGTAAAGTTATGGCGG	216	50	CCGCGCGATCGGCGATGAGACGT	465	51
CAGTAAAGTTATGGCGGCCGCGG	222	43	CCTACGCTGAATACCGCCATTCC	508	47
TAAAGTTATGGCGGCCGCGGCGG	225	43	CCGCCATTCCCGGCGACCCGAGA	521	53
CGAGATCAAGCCTGCCGATCTGG	294	50	CCATTCCCGGCGACCCGAGAGAC	524	53
ATTGCCGAAAAACACGTCAACGG	331	49	CCCGGCGACCCGAGAGACACCAC	529	53
CGTCAACGGCACAATGACGCTGG	345	50	CCGGCGACCCGAGAGACACCACC	530	53
GACGCTGGCAGAACTGAGCGCGG	360	46	CCCGAGAGACACCACCACGCCGC	537	52
AACAAATTGATTGCCCAGCTCGG	415	53	CCGAGAGACACCACCACGCCGCG	538	52
AAATTGATTGCCCAGCTCGGTGG	418	53	CCACCACGCCGCGGGCGATGGCG	548	48
GATTGCCCAGCTCGGTGGCCCGG	423	52	CCACGCCGCGGGCGATGGCGCAG	551	47
ATTGCCCAGCTCGGTGGCCCGGG	424	51	CCGCGGGCGATGGCGCAGACGTT	556	47
GCCCAGCTCGGTGGCCCGGGAGG	427	51	CCCAGCGGGCGCAGTTGGTGACG	614	47
CGGTGGCCCGGGAGGCGTGACGG	435	51	CCAGCGGGCGCAGTTGGTGACGT	615	47
ACGGCTTTTGCCCGCGCGATCGG	454	51	CCGGCGCAGCCAGCATTCGGGCC	656	48
CGGCGATGAGACGTTTCGTCTGG	474	53	CCAGCATTCGGGCCGGCTTACCG	665	48
ACGCTGAATACCGCCATTCCCGG	511	52	CCGGCTTACCGACGTCGTGGACT	677	50
CGAGAGACACCACCACGCCGCGG	539	52	CCGACGTCGTGGACTGTGGGTGA	685	47
GAGAGACACCACCACGCCGCGGG	540	52	CCGGCAGCGGCGACTACGGCACC	713	39
CACCACCACGCCGCGGGCGATGG	546	51	CCACCAATGATATTGCGGTGATC	734	49
GACGTTGCGTCAGCTTACGCTGG	573	50	CCAATGATATTGCGGTGATCTGG	737	49
ACGTTGCGTCAGCTTACGCTGGG	574	50	CCGCAGGGTCGTGCGCCGCTGGT	760	48
GCTTACGCTGGGTCATGCGCTGG	585	50	CCGCTGGTTCTGGTGACCTATTT	775	49
CTTACGCTGGGTCATGCGCTGGG	586	50	CCTATTTTACCCAGCCGCAACAG	791	48
ATGCGCTGGGCGAAACCCAGCGG	599	50	CCCAGCCGCAACAGAACGCAGAG	800	48
TGCGCTGGGCGAAACCCAGCGGG	600	50	CCAGCCGCAACAGAACGCAGAGA	801	42
CGAAACCCAGCGGGCGCAGTTGG	609	48	CCGCAACAGAACGCAGAGAGCCG	805	40
AGCGGGCGCAGTTGGTGACGTGG	617	47	CCGCCGCGATGTGCTGGCTTCAG	825	47
CAGTTGGTGACGTGGCTCAAAGG	625	49	CCGCGATGTGCTGGCTTCAGCGG	828	47
TGGCTCAAAGGCAATACGACCGG	637	49			
CGACCGGCGCAGCCAGCATTCGG	653	49			
GACCGGCGCAGCCAGCATTCGGG	654	49			
GGCGCAGCCAGCATTCGGGCCGG	658	48			
GGGCCGGCTTACCGACGTCGTGG	674	50			
CTTACCGACGTCGTGGACTGTGG	681	47			
TTACCGACGTCGTGGACTGTGGG	682	47			
TGGACTGTGGGTGATAAGACCGG	694	46			
GTGGGTGATAAGACCGGCAGCGG	700	46			
AAGACCGGCAGCGGCGACTACGG	709	38			
CGGCACCACCAATGATATTGCGG	729	49			
CCAATGATATTGCGGTGATCTGG	737	49			
TATTGCGGTGATCTGGCCGCAGG	744	49			
ATTGCGGTGATCTGGCCGCAGGG	745	49			
GCCGCAGGGTCGTGCGCCGCTGG	759	48			
GGGTCGTGCGCCGCTGGTTCTGG	765	48			
AGAGAGCCGCCGCGATGTGCTGG	819	40			
CCGCGATGTGCTGGCTTCAGCGG	828	47			
GCGGCGAGAATCATCGCCGAAGG	847	48			
CGGCGAGAATCATCGCCGAAGGG	848	48			

[a]Number of perfect matches of crRNA sequence with all genes from the $bla_{CTX\text{-}M}$ gene family.

S.T.2 bla$_{\text{CTX-M-15}}$ crRNA sequences used for in silico analysis of the bla$_{\text{CTX-M}}$ gene family

Sequence on sense strand (5' to 3')	Start	Hits[a]	Sequence on antisense strand (5' to 3')	Start	Hits[a]
ACTGCGCCAGTTCACGCTGATGG	15	45	CCAGTTCACGCTGATGGCGACGG	21	47
CCAGTTCACGCTGATGGCGACGG	21	47	CCGTCACGCTGTTGTTAGGAAGT	47	59
GCAACCGTCACGCTGTTGTTAGG	43	55	CCGCTGTATGCGCAAACGGCGGA	73	53
TGTGCCGCTGTATGCGCAAACGG	69	57	CCGAATTAGAGCGGCAGTCGGGA	113	56
GCCGCTGTATGCGCAAACGGCGG	72	53	CCAGTAAAGTGATGGCCGCGGCC	221	42
AAAAACTTGCCGAATTAGAGCGG	104	56	CCGCGGCCGCGGTGCTGAAGAAA	236	41
TGCCGAATTAGAGCGGCAGTCGG	111	56	CCGCGGTGCTGAAGAAAAGTGAA	242	59
GCCGAATTAGAGCGGCAGTCGGG	112	56	CCGAATCTGTTAAATCAGCGAGT	271	55
GAATTAGAGCGGCAGTCGGGAGG	115	56	CCTTGTTAACTATAATCCGATTG	312	39
GCGGCAGTCGGGAGGCAGACTGG	123	45	CCGATTGCGGAAAAGCACGTCAA	328	47
CGGCAGTCGGGAGGCAGACTGGG	124	45	CCGCGCTACAGTACAGCGATAAC	383	57
GTCGGGAGGCAGACTGGGTGTGG	129	43	CCCGGCTAGCGTCACCGCGTTCG	441	55
GTGCAGCACCAGTAAAGTGATGG	213	68	CCGGCTAGCGTCACCGCGTTCGC	442	55
CACCAGTAAAGTGATGGCCGCGG	219	42	CCGCGTTCGCCCGACAGCTGGGA	455	58
TAAAGTGATGGCCGCGGCCGCGG	225	42	CCCGACAGCTGGGAGACGAAACG	464	57
TGTTAACTATAATCCGATTGCGG	315	38	CCGACAGCTGGGAGACGAAACGT	465	57
ATTGCGGAAAAGCACGTCAATGG	331	47	CCGTCTCGACCGTACCGAGCCGA	489	47
TTGCGGAAAAGCACGTCAATGGG	332	47	CCGTACCGAGCCGACGTTAAACA	498	47
CGTCAATGGGACGATGTCACTGG	345	49	CCGAGCCGACGTTAAACACCGCC	503	49
GTCACTGGCTGAGCTTAGCGCGG	360	52	CCGACGTTAAACACCGCCATTCC	508	50
GCTACAGTACAGCGATAACGTGG	387	57	CCGCCATTCCGGGCGATCCGCGT	521	51
AATAAGCTGATTGCTCACGTTGG	415	51	CCATTCCGGGCGATCCGCGTGAT	524	52
AAGCTGATTGCTCACGTTGGCGG	418	51	CCGGGCGATCCGCGTGATACCAC	529	52
GATTGCTCACGTTGGCGGCCCGG	423	52	CCGCGTGATACCACTTCACCTCG	538	57
CACCGCGTTCGCCCGACAGCTGG	453	55	CCACTTCACCTCGGGCAATGGCG	548	57
ACCGCGTTCGCCCGACAGCTGGG	454	58	CCTCGGGCAATGGCGCAAACTCT	556	53
GACGTTAAACACCGCCATTCCGG	510	52	CCAACGGGCGCAGCTGGTGACAT	615	55
ACGTTAAACACCGCCATTCCGGG	511	52	CCACCGGTGCAGCGAGCATTCAG	653	54
CGCGTGATACCACTTCACCTCGG	539	57	CCGGTGCAGCGAGCATTCAGGCT	656	48
GCGTGATACCACTTCACCTCGGG	540	58	CCTGCTTCCTGGGTTGTGGGGGA	685	60
TACCACTTCACCTCGGGCAATGG	546	57	CCTGGGTTGTGGGGGATAAAACC	692	61
GGGCAATGGCGCAAACTCTGCGG	560	47	CCGGCAGCGGTGGCTATGGCACC	713	32
AACTCTGCGGAATCTGACGCTGG	573	48	CCACCAACGATATCGCGGTGATC	734	49
ACTCTGCGGAATCTGACGCTGGG	574	48	CCAACGATATCGCGGTGATCTGG	737	49
TCTGACGCTGGGTAAAGCATTGG	585	59	CCAAAAGATCGTGCGCCGCTGAT	760	60
CTGACGCTGGGTAAAGCATTGGG	586	59	CCGCTGATTCTGGTCACTTACTT	775	59
AAGCATTGGGCGACAGCCAACGG	599	47	CCCAGCCTCAACCTAAGGCAGAA	800	56
AGCATTGGGCGACAGCCAACGGG	600	47	CCAGCCTCAACCTAAGGCAGAAA	801	56
CGACAGCCAACGGGCGCAGCTGG	609	48	CCTCAACCTAAGGCAGAAAGCCG	805	56
AACGGGCGCAGCTGGTGACATGG	617	55	CCTAAGGCAGAAAGCCGTCGCGA	811	57
CAGCTGGTGACATGGATGAAAGG	625	55	CCGTCGCGATGTATTAGCGTCGG	825	55
TGGATGAAAGGCAATACCACCGG	637	54			
CACCGGTGCAGCGAGCATTCAGG	654	54			
GGTGCAGCGAGCATTCAGGCTGG	658	49			
AGGCTGGACTGCCTGCTTCCTGG	674	52			
GGCTGGACTGCCTGCTTCCTGGG	675	53			
ACTGCCTGCTTCCTGGGTTGTGG	681	60			
CTGCCTGCTTCCTGGGTTGTGGG	682	60			
TGCCTGCTTCCTGGGTTGTGGGG	683	60			
GCCTGCTTCCTGGGTTGTGGGGG	684	60			
TGGGTTGTGGGGGATAAAACCGG	694	61			
GTGGGGGATAAAACCGGCAGCGG	700	58			
GGGGATAAAACCGGCAGCGGTGG	703	32			
AAAACCGGCAGCGGTGGCTATGG	709	32			
TGGCACCACCAACGATATCGCGG	729	73			
CCAACGATATCGCGGTGATCTGG	737	49			
AGATCGTGCGCCGCTGATTCTGG	765	60			
CTTCACCCAGCCTCAACCTAAGG	795	56			
CCGTCGCGATGTATTAGCGTCGG	825	55			
TCGCGATGTATTAGCGTCGGCGG	828	54			
GCGGCTAAAATCGTCACCGACGG	847	47			

[a]Number of perfect matches of crRNA sequence with all genes from the bla$_{\text{CTX-M}}$ gene family.

S.T.3 crRNA sequences used for gene targeting

Targeted gene/gene family	Starting position[a]	sequence
$bla_{CTX\text{-}M\text{-}15}$	111+	5' TGCCGAATTAGAGCGGCAGT 3'
$bla_{CTX\text{-}M\text{-}14}$	819+	5' AGAGAGCCGCCGCGATGTGC 3'
RepA2	796+	5' GATTACACTGAGTTTAAACG 3'
bla_{KPC}	648+	5' CAACCACCGCATCCGCGCGG 3'
bla_{NDM}	381+	5' CGGTATGGACGCGCTGCATG 3'
bla_{NDM}*(2)*	53+	5' CCGCTGCATTGATGCTGAGC 3'

[a]The +-sign indicates that the crRNA targets the sense strand.

References

[1] David, Herbert Aron, and Haikady Navada Nagaraja. "Order statistics." John Wiley & Sons, Inc., 1981.

[2] Ewens, Warren J., and Herbert S. Wilf. "Computing the distribution of the maximum in balls-and-boxes problems with application to clusters of disease cases." Proceedings of the National Academy of Sciences 104.27 (2007): 11189-11191.

[3] Ekhad, Shalosh B., and Doron Zeilberger. "Balls in Boxes: Variations on a Theme of Warren Ewens and Herbert Wilf." Advances in Combinatorics. Springer Berlin Heidelberg, 2013. 161-174.
